# Effect of different cooking methods on the content of total vitamin C, ascorbic acid and dehydroascorbic acid of the galega kale

**DOI:** 10.1080/07853890.2021.1896080

**Published:** 2021-09-28

**Authors:** Carolina Vieira, Marta da Silva, Margarida Simões, Gonçalo Rodrigues, Tânia Albuquerque, Renata Ramalho, Paula Pereira

**Affiliations:** a Instituto Universitário Egas Moniz (IUEM), Egas Moniz Cooperativa de Ensino Superior, Caparica, Portugal; b Department of Food and Nutrition, National Institute of Health Dr. Ricardo Jorge (INSA), Lisbon, Portugal

## Abstract

**Introduction:**

Adequate daily intake of vitamin C is essential to maintain numerous physiological processes in the human body. Humans cannot produce endogenous vitamin C and therefore are dependent on dietary intake [1]. Galega kale is grown and consumed across the world and is a rich source of antioxidants such as vitamin C [2]. However, little is known about how domestic cooking methods affect the nutritional composition and bioavailability of nutrients in fresh vegetables. The aim of this study was to investigate the effect of four different domestic cooking methods (boiling, braising, sautéing and a typical Portuguese soup, caldo-verde) on the content of total vitamin C, ascorbic acid and dehydroascorbic acid of the galega kale.

**Materials and methods:**

Samples of galega kale were obtained from the University Garden of Instituto Universitário Egas Moniz. Samples were washed and the inedible parts were discarded. The final samples consisted of 16 g of kale with a ratio of leaves to stems of 75/25. Samples were submitted to four domestic cooking methods: boiling, sautéing, braising and a traditional Portuguese soup, caldo-verde and raw Galega Kale was used as a control. Determination of total vitamin C, acid ascorbic (AA) and acid dehydroascorbic (DHA) was done using high-performance liquid chromatography (HPLC) according to the method described by Valente et al. [3].

**Results:**

The caldo-verde soup led to the greatest reduction in total vitamin C content (–77%), followed by boiling (–63%), sautéing (–48%) and braising (–29%). AA content decreased by 65%, 38% and 22% for caldo-verde, boiling and sautéing, respectively, whereas, braising increased AA by 16%. Sautéing and braising showed comparable effects on DHA content (–71% and −72%, respectively), while caldo-verde and boiling had a greater impact (–88% and −85%, respectively). **Discussion and conclusions:** Results of this study show that cooking techniques can negatively affect the vitamin C content of galega kale. The extent of this reduction was dependent on the cooking technique, temperature and duration used. The greatest reductions in total vitamin C, AA and DHA were seen when water was used as a source of heat (i.e. boiling and caldo-verde). The longer boiling period (∼75 min) used for the caldo-verde soup likely lead to the greater reduction in vitamin C when compared to boiling (10 min). These effects are consistent with observations done by others [2,4]. Our findings suggest that sautéing and braising are the two cooking techniques that have the least detrimental effect on vitamin C content in galega kale.
